# Interactions of Alkylphosphocholines with Model Membranes—The Langmuir Monolayer Study

**DOI:** 10.1007/s00232-013-9557-4

**Published:** 2013-05-15

**Authors:** Anita Wnętrzak, Kazimierz Łątka, Patrycja Dynarowicz-Łątka

**Affiliations:** 1 Institute of Physics, Jagiellonian University, Reymonta 4, 30-059 Kraków, Poland; 2 Faculty of Chemistry, Jagiellonian University, Ingardena 3, 30-060 Kraków, Poland

**Keywords:** Alkylphosphocholine, Model membrane, Langmuir monolayer, Interaction

## Abstract

Alkylphosphocholines (APCs) belong to a class of synthetic antitumor lipids, which are new-generation anticancer agents. In contrast to traditional antitumor drugs, they do not attack the cell nucleus but, rather, the cellular membrane; however, their mechanism of action is not fully understood. This work compared the interactions of selected APCs [namely, hexadecylphosphocholine (miltefosine), octadecylphosphocholine and erucylphosphocholine] with the most important membrane lipids [cholesterol, 1,2-dipalmitoyl-*sn*-glycero-3-phosphocholine (DPPC) and 1-palmitoyl-2-oleoyl-*sn*-glycero-3-phosphocholine (POPC)] and examined their influence on a model membrane of tumor and normal cells. As a simple model of membranes, Langmuir monolayers prepared by mixing cholesterol either with a saturated phosphatidylcholine (DPPC), for a normal cell membrane, or with an unsaturated one (POPC), for a tumor cell membrane, have been applied. The APC–lipid interactions, based on experimental surface pressure (*π*) versus mean molecular area (A) isotherms, were analyzed qualitatively (with mean molecular area values) as well as quantitatively (with the Δ*G* ^exc^ function). Strong attractive interactions were observed for mixtures of APCs with cholesterol, contrary to the investigated phosphatidylcholines, for which the interactions were found to be weak with a tendency to separation of film components. In ternary monolayers it has been found that the investigated model systems (cholesterol/DPPC/APC vs cholesterol/POPC/APC) differ significantly as regards the interactions between film-forming molecules. The results demonstrate stronger interactions between the components of cholesterol/POPC/APC monolayers compared to cholesterol/POPC film, mimicking tumor cell membranes. In contrast, the interactions in cholesterol/DPPC/APC films were found to be weaker than those in the cholesterol/DPPC system, serving as a model of healthy cell membranes, thus proving that the incorporation of APCs is, from a thermodynamic point of view, unfavorable for binary cholesterol/DPPC monolayers. It can be concluded that the composition of healthy cell membranes is a natural barrier preventing the incorporation of APCs into normal cells.

## Introduction

To gain insight into the mechanism of physiological activity (mode of action, selectivity, toxicity) of biomolecules acting at the cellular membrane level, different methods can be applied, which are usually based on examining the interactions between a bioactive molecule and cellular membrane components. These interactions can be studied in natural systems, either isolated or not (living cells), or in membrane models. The former are highly variable and complicated and, thus, provide only a general view on a particular problem of interest, while the latter have the advantage of being simple and well-defined, therefore enabling the study of a specific aspect of a given phenomenon (Maget-Dana 1999).

Many different membrane models (reviewed in Peetla et al. 2009; Chan and Boxer 2007) have been applied to investigate the interactions between biochemicals and membrane components. Most popular are Langmuir monolayers (Peetla et al. 2009), liposomes or vesicles (Kell 1981), black lipid membranes (Ottova and Tien 1997) and surface-confined membrane systems (Richter et al. 2006). Although none of these models is perfect and fully universal (advantages and disadvantages of using the above-mentioned membrane models are discussed in Hąc-Wydro and Dynarowicz-Łątka 2008), the Langmuir monolayer technique, the principles of which can be found elsewhere (Gaines 1966), is a potent and frequently applied method for mimicking cellular membranes (Maget-Dana 1999; Hąc-Wydro and Dynarowicz-Łątka 2008; Brockman 1999) and very useful to study biomolecule–membrane interactions. These interactions can be considered crucial for understanding the mode of action of drugs acting on the membrane level (Maget-Dana 1999; Hąc-Wydro and Dynarowicz-Łątka 2008).

**Fig. 1:**
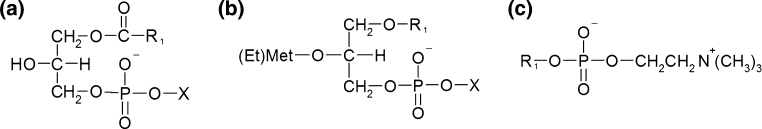
General chemical structures of **a** lysophosphatidylcholine (LPC), **b** alkyllysophospholipid (ALP) and **c** alkylphosphocholine (APC)

**Fig. 2:**
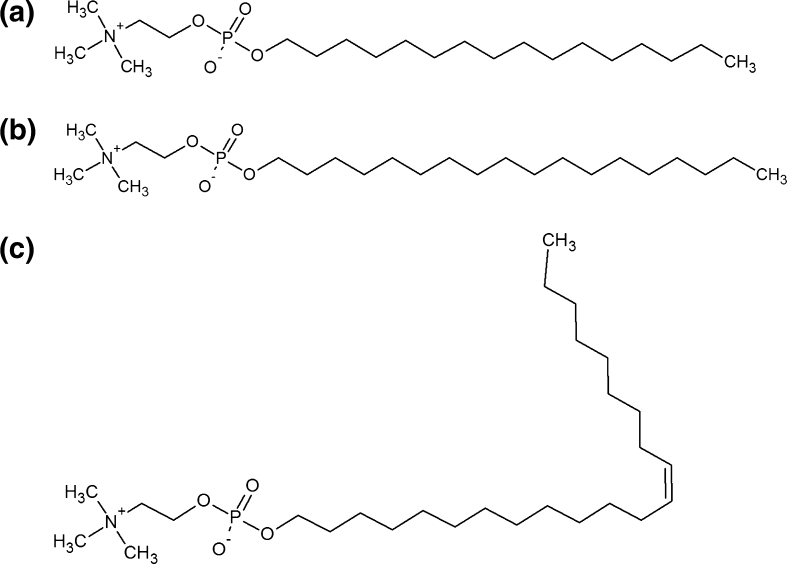
General chemical structures of **a** hexadecylphosphocholine (HePC), **b** octadecylphosphocholine (OcPC) and **c** erucylphosphocholine (ErPC)

A good example of membrane-targeted drugs are synthetic analogues of lysophosphatidylcholine (Fig. 1a), generally termed alkyllysophospholipids (Gajate and Mollinedo 2002) (Fig. 1b), known for their anticancer properties. Attempts to find the minimal moiety in the phospholipid structure that maintains antitumor properties have led to the discovery of alkylphosphocholines (APCs) (Fig. 1c) (Eibl et al. 1992) and subsequent synthesis of their first homologue, hexadecylphosphocholine (HePC, miltefosine) (Fig. 2a) (Eibl and Unger 1990).

The main disadvantage of using this drug was its inapplicability in oral administration due to severe gastrointestinal side effects and hemolysis upon intravenous injections. In order to overcome these problems, chemical modifications in the structure of APCs have been made; i.e., the hydrophobic part was modified either by its elongation [as exemplified by octadecylphosphocholine (OcPC) (Fig. 2b)] and/or by introduction of a double bond [as exemplified by erucylphosphocholine (ErPC) (Fig. 2c)] (Berger et al. 1998; Van der Luit et al. 2007; Rübel et al. 2006).

These membrane-targeted drugs, before affecting vital functions inside the cell—such as cell proliferation, cell cycle progression, differentiation, invasion and angiogenesis (Takai et al. 2008)—have to penetrate through the biomembrane. So far, little is known about how APCs interact with membrane constituents. Also, it is still unclear which membrane component is responsible for the high selectivity of APCs, i.e., targets the drug molecule to the tumor cell membrane, sparing the normal cells. To gain a deeper insight into these issues, it is of utmost importance to (1) examine systematically the interactions between the drugs and cellular membrane components and (2) compare the effect of APCs on model tumors and healthy cell membranes.

For this purpose, in the first step of our investigations, we selected three main membrane lipids [i.e., cholesterol, 1,2-dipalmitoyl-*sn*-glycero-3-phosphocholine (DPPC) and 1-palmitoyl-2-oleoyl-*sn*-glycero-3-phosphocholine (POPC)] to study their interactions with selected APCs, having the same polar part and differing in the length of the hydrocarbon chain and saturation degree, namely, HePC, OcPC and ErPC. Cholesterol was chosen because of its crucial role in regulating membrane physicochemical properties in eukaryotic cells (Crane and Tamm 2004) as well as its involvement in the formation of ordered lipid rafts (Fan et al. 2010), which have been hypothesized to be a site of action of synthetic antitumor lipids (Heczkova and Slotte 2006; Van der Luit et al. 2007). DPPC and POPC, on the other hand, are the most abundant phospholipids building the biomembranes; and their proportion significantly differs in normal versus tumor membranes (Agatha et al. 2001). It is known that the membrane of healthy cells contains a higher amount of cholesterol and saturated phospholipids, whereas tumor cellular membrane is characterized by the presence of unsaturated lipids in higher amounts (Klock and Pieprzyk 1979). Therefore, the cancer cell membrane is more fluid compared to the normal cell membrane (Inbar et al. 1977). With the Langmuir monolayer technique it is possible to model cell membranes of healthy and cancerous cells and monitor changes in membrane properties caused by the addition of the investigated APCs, which was done in the second part of our study. For our research we chose leukemic cell membranes, composed of cholesterol and POPC, and normal cellular leucocyte membranes, containing cholesterol and DPPC.

We believe that our investigations will have lead to a better understanding of the membrane activity of alkylphospholipids, one of the most interesting and efficient antineoplastic agents.

## Materials and Methods

The following materials were purchased and used: OcPC (AG Scientific, San Diego, USA), HePC (Avanti Polar Lipids, Alabaster, USA), cholesterol (Sigma, St. Louis, USA) and POPC and DPPC (Avanti Polar Lipids, Alabaster, USA). All of these products were of high purity (>99 %) and used as received. ErPC was kindly supplied by Aeterna Zentaris GmbH (Frankfurt, Germany). The investigated compounds were dissolved in a chloroform:methanol (Sigma-Aldrich, St. Louis, USA) 9:1 v/v mixture with a typical concentration of 0.2–0.5 mg/ml. Mixed solutions were obtained by mixing proper volumes of the respective stock solutions. Membrane models of leukemic and healthy cells were prepared based on Tsuchiya et al. (2002). In order to model the normal leucocyte cell membrane, cholesterol and DPPC were mixed in a proportion of 0.67, while tumor cell membrane was formed by mixing cholesterol with POPC in a ratio of 0.25.

The experiments were performed using Langmuir trough (total area = 600 cm^2^) placed on an antivibration table. Surface pressure (*π*) was measured with an accuracy of ±0.1 mN/m using a Wilhelmy plate made of chromatography paper (Whatman Chr1; Whatman, Piscataway, NJ). Mixtures containing APCs (mole fraction *X*_APCs_ = 0.1–0.9) were prepared from stock solutions. Mixed monolayers were obtained by dropping the spreading solutions onto the water surface (ultrapure water produced by a Nanopure water purification system [APS Water, Lake Balboa, CA] coupled to a Milli-Q water purification system, resistivity = 18.2 MΩ cm) using a microsyringe (Hamilton, Reno, NV). The average number of molecules spread onto the water surface (pH 5.6) in a single experiment was ca. 4.54 × 10^16^. All experiments were performed at 20 °C. The subphase temperature was controlled to within 0.1 °C by a circulating water system from Julabo (Allentown, PA). After spreading, 10 min was allowed for the solvent to evaporate. Afterward compression was initiated with a barrier speed of 11 Å^2^/(molecule min). Each experiment was repeated two or three times to ensure high reproducibility of the obtained isotherms to ±2 Å^2^.

The above-mentioned conditions were chosen as optimal for performing Langmuir monolayer experiments for the studied APCs. However, one has to be aware of the potential errors resulting from using a Teflon barrier, spreading solvent (chloroform) of greater density than water and use of a Wilhelmy plate made of filter paper (for details, see Brzozowska and Figaszewski 2002).

## Results

### Isotherms of Pure Components

In the first step of our studies, three investigated APCs (HePC, OcPC, ErPC) and selected membrane lipids (cholesterol, DPPC, POPC) were studied alone in Langmuir monolayers.

Regarding the isotherms for pure membrane lipids, their characteristics are very well known and our results are in a good agreement with the data already published (see, e.g., Cadena-Nava et al. 2006 for cholesterol; for DPPC Klopfer and Vanderlick 1996; Duncan and Larson 2008; Crane et al. 1999; and for POPC Yun et al. 2003). Cholesterol forms very condensed monolayers and collapses at a surface pressure of about 44 mN/m, while DPPC exhibits a characteristic transition region (at ca. 5 mN/m), ascribed to orientation changes of molecules upon compression, and collapses at ca. 63 mN/m. On the other hand, POPC forms a liquid-expanded monolayer without any visible transition in the course of the isotherm and has a lower collapse pressure (50 mN/m) compared to the saturated phospholipid (DPPC).

**Fig. 3:**
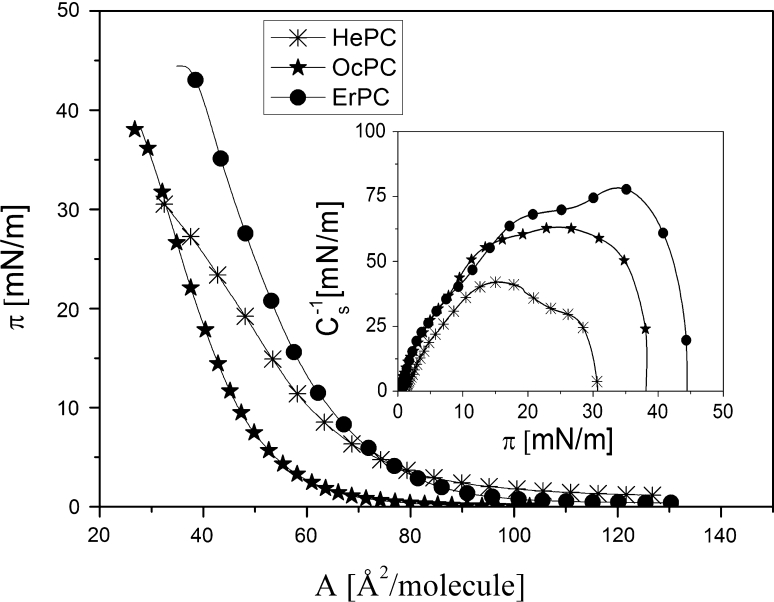
Surface pressure (*π*)–area (*A*) isotherms of HePc, OcPC and ErPC spread at the air/water interface at 20 °C. Inset Compression modulus (*C*_s_^−1^)–surface pressure (*π*) dependencies

Figure 3 confirms that the studied APCs are capable of forming monomolecular layers at the free water surface. They all form liquid-type films, as proved by the compression moduli *C*_S_^−1^ = −*A* (d*π*/d*A*) (Davies and Rideal 1963) values, shown in the inset. Comparing maximum *C*_S_^−1^ values, it is evident that upon increasing of the hydrocarbon chain length, the monolayer successively becomes more condensed. Another characteristic feature is that upon elongation of the hydrophobic part of the APC molecule, the film becomes more stable, as indicated by higher values of collapse pressures (*π*_coll_). All of the characteristic parameters for the investigated APC monolayers are summarized in Table 1.

**Table 1:** Characteristic parameters of HePC, OcPC and ErPC monolayers recorded at 20 °C

	“Lift-off” (Å^2^/molecule)	*A*_0_ (Å^2^/molecule)	*π*_coll_ (mN/m)	*A*_coll_ (Å^2^/molecule)	*C*_s_^−1^_max_ (mN/m)
HePC	98	78	31	32	41
OcPC	70	68	38	28	63
ErPC	93	75	44	40	78

### Two-Component Monolayers

Since the investigated APCs were proved to form floating monolayers on aqueous subphases, the Langmuir monolayer technique can be applied for studying their interactions with membrane lipids.

**Fig. 4:**
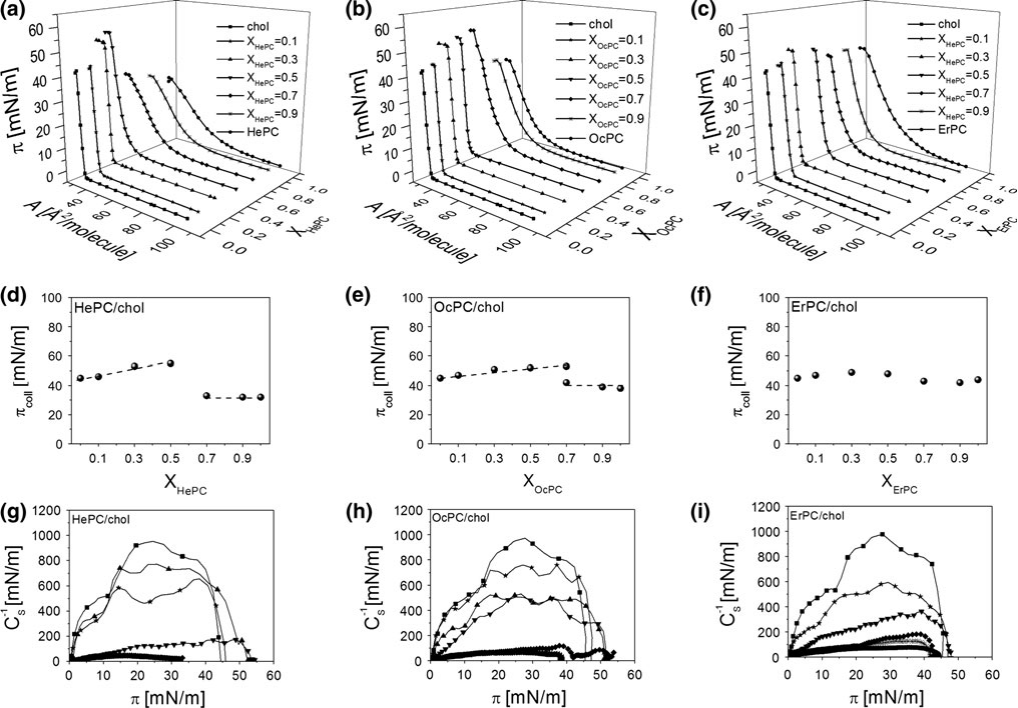
Surface pressure (*π*)–area (*A*) isotherms for APCs, cholesterol and their mixtures (**a**–**c**). Collapse pressure (*π*_coll_) versus mixed film composition (X_APCs_) plots (**d**–**f**). Compression modulus (*C*_s_^−1^)–surface pressure (*π*) dependencies (**g**–**i**)

**Fig. 5:**
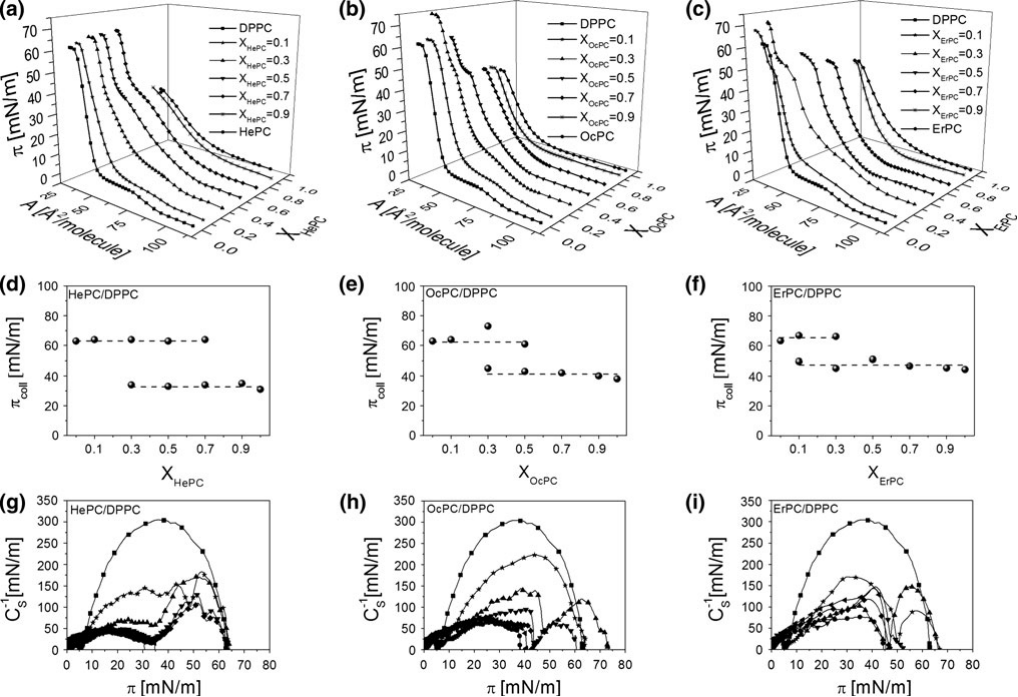
Surface pressure (*π*)–area (A) isotherms for APCs, DPPC and their mixtures (**a**–**c**). Collapse pressure (*π*_coll_) versus mixed film composition (X_APCs_) plots (**d**–**f**). Compression modulus (*C*_s_^−1^)–surface pressure (*π*) dependencies (**g**–**i**)

**Fig. 6:**
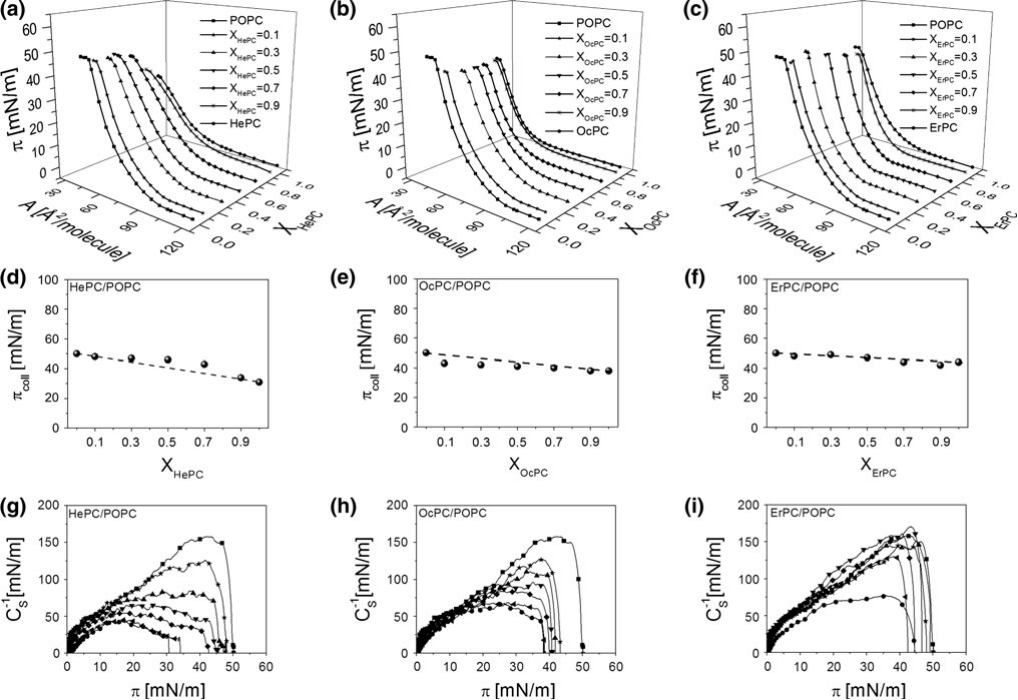
Surface pressure (*π*)–area (A) isotherms for APCs, POPC and their mixtures (**a**–**c**). Collapse pressure (*π*_coll_) versus mixed film composition (X_APCs_) plots (**d**–**f**). Compression modulus (*C*_s_^−1^)–surface pressure (*π*) dependencies (**g**–**i**)

Monolayers of HePC, OcPC and ErPC mixed with membrane lipids (cholesterol, DPPC and POPC) were prepared for five different mole fractions of APCs (*X*_APC_): 0.1, 0.3, 0.5, 0.7 and 0.9. The resulting isotherms are shown in Figs. 4, 5, 6a–c.

Let us first discuss mixed systems with cholesterol (Fig. 4). Looking at the isotherms, it is seen that cholesterol exerts a condensing effect on monolayers from all the studied APCs. The *π*–A isotherms of pure cholesterol and *X*_APC_ = 0.1 are very similar, while those for *X*_APC_ = 0.3 and 0.5 have their lift-off areas shifted toward smaller values in respect to the monolayer of cholesterol. All of the remaining mixed isotherms of a higher proportion of APCs are similar in shape to the respective APCs; however, they are shifted toward smaller areas in respect to pure antitumor lipids.

To characterize the physical state of the investigated monolayers, compression moduli values were calculated from the isotherm data points and plotted as a function of surface pressure (Fig. 4g–i). The highest values of compression moduli, corresponding to the maxima on the *C*_S_^−1^ *=* *f*(*π*) curves, were reached for pure cholesterol (ca. 1,000 mN/m) and mixed monolayers of *X*_APC_ = 0.1–0.3, which are all of solid type. On the other hand, pure monolayers of APCs as well as mixtures containing an excess of APCs form liquid-type films.

To gain insight into the miscibility of APC/cholesterol systems, collapse pressure values have been plotted versus film composition (Fig. 4d–f). According to the phase rule, if two components are miscible in monolayers, the collapse pressure varies with mixed film composition (Crisp 1949; Wu and Huntsberger 1969). For HePC and OcPC the behavior is similar; i.e., cholesterol-rich monolayers can be clearly identified as miscible as their collapse pressures vary with film composition, contrary to films containing HePC or OcPC in excess (*X* ≥ 0.7), for which the collapse pressure remains constant. Interestingly, for a mixture containing *X*_OcPC_ = 0.7, two collapses appear in the course of the isotherm. The first collapse occurs at a pressure close to the value of *π*_coll_ for pure OcPC, while the other occurs at an elevated pressure (53 mN/m). In general, the presence of two independent collapses in the course of an isotherm from two film-forming molecules, corresponding to collapse pressure values for pure components, is evidence of their immiscibility in a monolayer (Dynarowicz-Łątka and Kita 1999). The first collapse corresponds to the ejection of a substance collapsing at lower pressures, while the other one collapses at higher pressure. Thus, for the discussed mixture of *X*_OcPC_ = 0.7 it is evident that at a lower collapse pressure OcPC is first expelled from the monolayer and afterward, at a higher pressure, the remaining component (cholesterol) collapses. Similar behavior of two collapses can be expected for *X*_OcPC_ = 0.9; however, the second collapse is not visible as it is expected to occur at a very low area (due to a small proportion of cholesterol in the mixed monolayer), which is out of the moving barrier range.

From analysis of the plots of *π*_coll_ versus film composition it may be deduced that the miscibility of HePC and OcPC with cholesterol depends on mixed film composition, i.e., for films rich in cholesterol both components mix in monolayers, contrary to films containing an excess of antitumor lipid, wherein film separation occurs.

For monolayers composed by ErPC and cholesterol, due to very similar values of collapse pressures of pure components and their mixtures, the conclusion regarding miscibility based on collapse pressure analysis is not as evident as in the former cases. It looks like the system is miscible within the whole range of mole fractions; however, further analysis based on mean molecular area values and excess free energy values is required for this particular mixture and will be discussed later.

In Fig. 5a–c the isotherms for mixtures with DPPC are presented. As can be seen, the course of the isotherm changes systematically upon addition of APCs, from the shape similar to pure DPPC to that characteristic for pure APC. For mixtures of *X*_APC _= 0.1, the plateau region characteristic for DPPC (reflecting the phase transition between liquid-expanded and liquid-condensed states) is observed. With the increase of APC content in a monolayer, this plateau region gradually shortens, shifts to higher pressures and finally disappears. A characteristic feature of mixed films of *X*_HePC_ = 0.3–0.7, *X*_OcPC_ = 0.3–0.5 and *X*_ErPC_ = 0.3 is the presence of two collapses in the course of the isotherms. From the collapse pressure values plotted versus film composition (Fig. 5d–f) it can be observed that both the first and the second collapse remain nearly constant upon changing the components’ proportion; the first collapse occurs at a surface pressure close to that for pure APC, while the second occurs at a similar pressure to that for pure DPPC. This may suggest immiscibility and phase separation in mixtures with DPPC.

Figure 6a–c shows the isotherms recorded for mixtures with POPC. In this case, mixed monolayers are situated between those obtained for the respective one-component films. The monolayers of both APCs and POPC as well as their mixtures are of liquid character. The isotherms lack any phase transition. The collapse pressure changes almost linearly with film composition for HePC-containing film (Fig. 6d). For the remaining systems, the collapse pressure values for mixtures with either OcPC or ErPC are very similar to that for pure POPC. Therefore, a further thermodynamic analysis is required to gain insight into the miscibility of components in these mixtures.

**Fig. 7:**
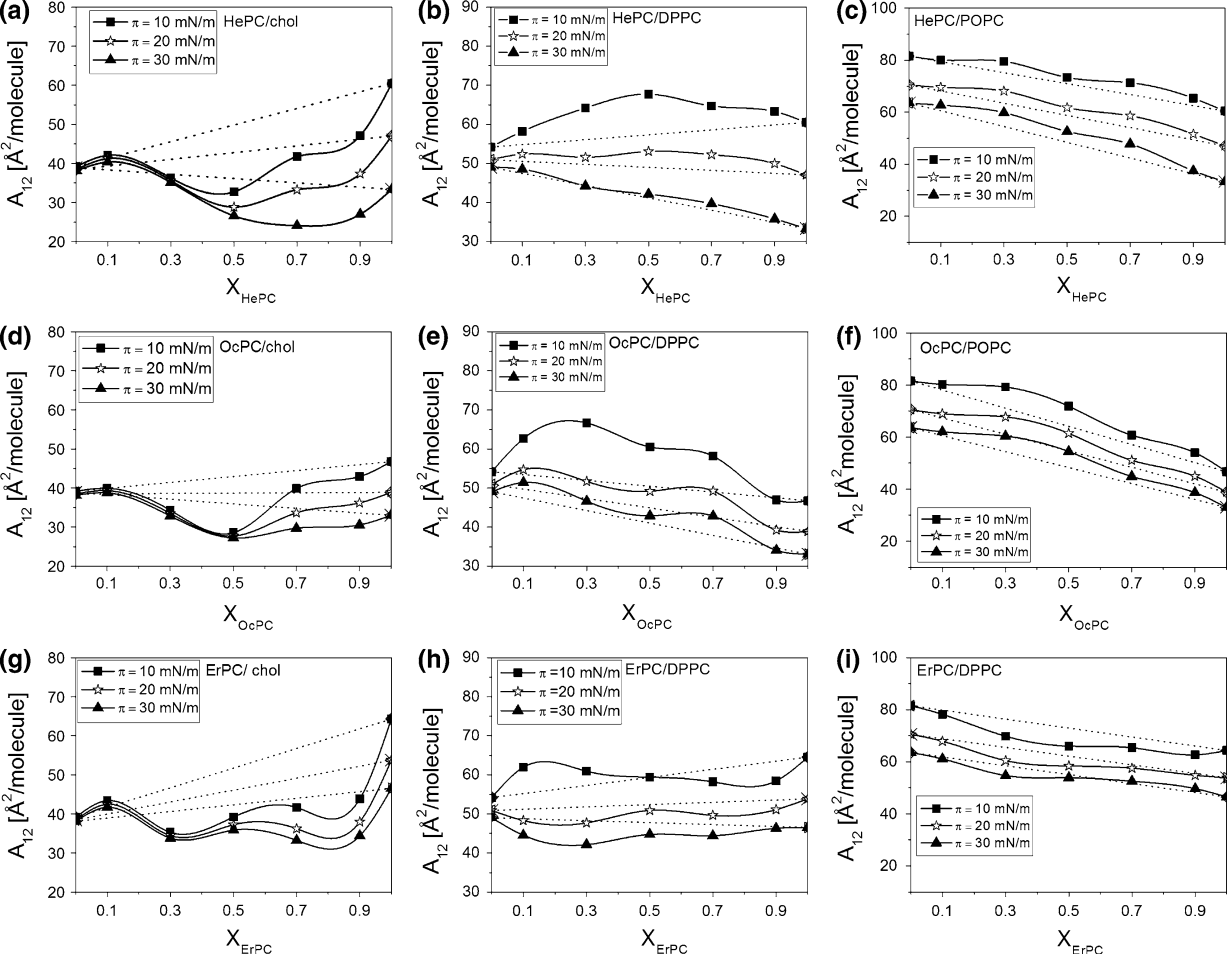
Mean molecular area (*A*_12_) versus mixed film composition (X_APCs_) plots for mixtures of APCs with investigated lipids at different constant surface pressures

For a qualitative analysis of the interactions between APCs and membrane lipids, the mean molecular area values (obtained directly from the isotherms) versus film composition have been plotted (Fig. 7).

For mixtures with cholesterol (Fig. 7a, d, g), negative deviations from ideal behavior are observed, indicating attractive interactions occurring between all of the investigated APCs and cholesterol. The extent of deviations is dependent on surface pressure and film composition. For HePC and OcPC/cholesterol mixtures, the most significant deviations are seen at a 1:1 proportion of the components, while for the ErPC/cholesterol system the strength of attractive interactions seems to be comparable for a broad composition range (X_ErPC_ = 0.3–0.9). Not only the film composition but also the surface pressure influences the strength of interactions; namely, at elevated surface pressures, the interactions are stronger, which is logical considering the closer molecular packing.

Different behavior is seen for mixtures with DPPC (Fig. 7b, e, h). Positive deviations, indicating repulsive interactions, confirm immiscibility and phase separation between molecules in HePC and OcPC/DPPC monolayers. For ErPC mixed with DPPC, small positive deviations appear only at low *π*, while at higher pressures slight negative deviations are seen. For mixtures with POPC (Fig. 7c, f, i), in general, the extent of interactions is smaller compared to DPPC (slightly repulsive interactions are observed for monolayers containing HePC and OcPC and attractive ones for ErPC).

### Thermodynamic Analysis of APC/Lipid Monolayers

**Fig. 8:**
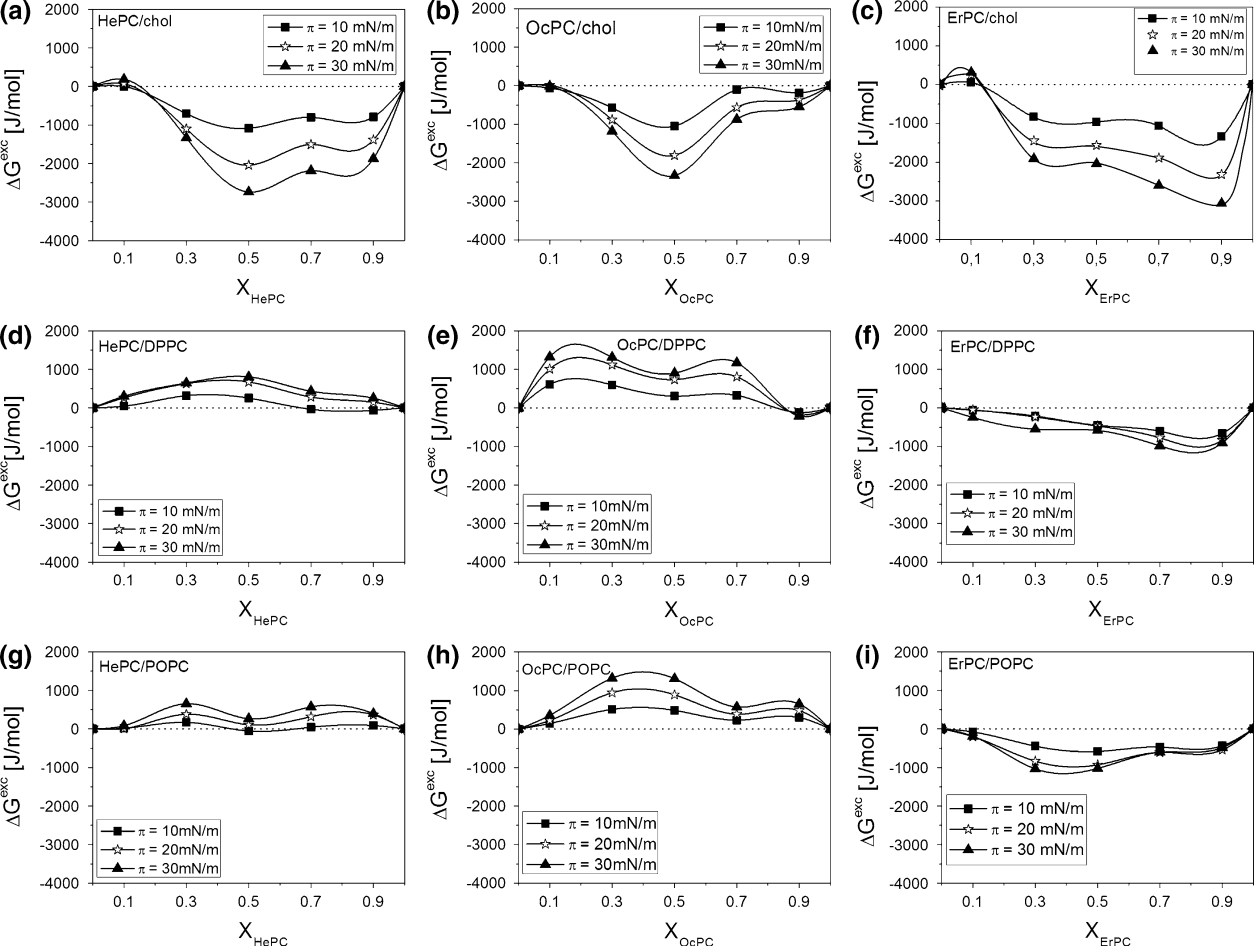
Excess free enthalpy of mixing (Δ*G* ^exc^) versus mixed film composition (X_APCs_) plots for mixtures of APCs with investigated lipids at different constant surface pressures

To quantify the above-mentioned interactions, values of the excess free enthalpy changes (Δ*G* ^exc^) at different *π* were calculated from the isotherm data points (Fig. 8) using the following equation (Pagano and Gershweld 1972)

1 $$ \Updelta G^{\text{exc}} = N_{A} \int\limits_{0}^{\pi } {A^{\text{exc}} {\text{d}}\pi } $$

where *A* ^exc^ is defined as the difference between the mean molecular area *A*_12_ observed at a given *π* value for a particular mole ratio of the components and the ideal mean molecular area *A*_12_^id^, which is defined as the weighted average of the mean molecular areas (*A*_1_ and *A*_2_) observed for the one-component monolayers at the same surface pressure (*A*_12_^id^ = *A*_1_ *X*_1_ + *A*_2_[1 − *X*_1_], where *X*_1_ is the mole ratio of component 1 in the binary film).

Strong negative values of Δ*G* ^exc^ for mixtures with cholesterol are indicative of potent attractive interactions between APCs and cholesterol molecules. The occurrence of a minimum implies that the influence of molecular interactions on film stability was most significant at that very composition. Such strong attractive interactions may result from the formation of surface complexes, as has been postulated by other authors (Seoane et al. 1998; Saint-Pierre-Chazalet et al. 1988; Gershfeld 1978; Albrecht et al. 1981; Gong et al. 2002) to interpret the deviations from ideality observed for a particular mixture composition. For the investigated HePC and OcPC mixtures with cholesterol, due to the strongest interactions at X_APC_ = 0.5, it may be postulated that APC–cholesterol complexes of 1:1 stoichiometry are formed at the surface. Further elongation of the hydrophobic part of the molecule together with the introduction of a double bond (ErPC) causes the interactions with cholesterol to be similar within a wide composition range and their strength gradually increases upon addition of ErPC into the film, without any clear minimum in the curve.

Differences in interactions of ErPC versus HePC and OcPC are more pronounced in mixtures with phospholipids. Independently of the degree of unsaturation of the hydrophobic chains of phosphatidylcholines (DPPC or POPC), both HePC and OcPC show repulsive interactions, in contrast to ErPC, for which small attractive intermolecular forces are observed. This proves that although the elongation of the hydrophobic chain length from C16 to C18 does not change much the behavior of APCs mixed with cholesterol or phospholipids, a further increase of the hydrocarbon chain length to C22 together with the presence of a double bond exerts a more significant change in the nature and strength of the interaction, especially when phospholipids are concerned.

### Influence of APCs on Model Membranes—Three-Component Monolayers

In order to gain insight into the influence of the investigated APCs on normal and tumor cells, their membranes were modeled with Langmuir monolayers composed of cholesterol and phosphatidylcholines (DPPC or POPC) in the proportion specified above (“Materials and Methods” section). The investigated APCs were added into both model membranes in various concentrations (*X*_APC_ = 0, 0.1, 0.3, 0.5, 0.7, 0.9, 1), keeping the cholesterol/phosphatidylcholine proportion in the respective model systems constant.

**Fig. 9:**
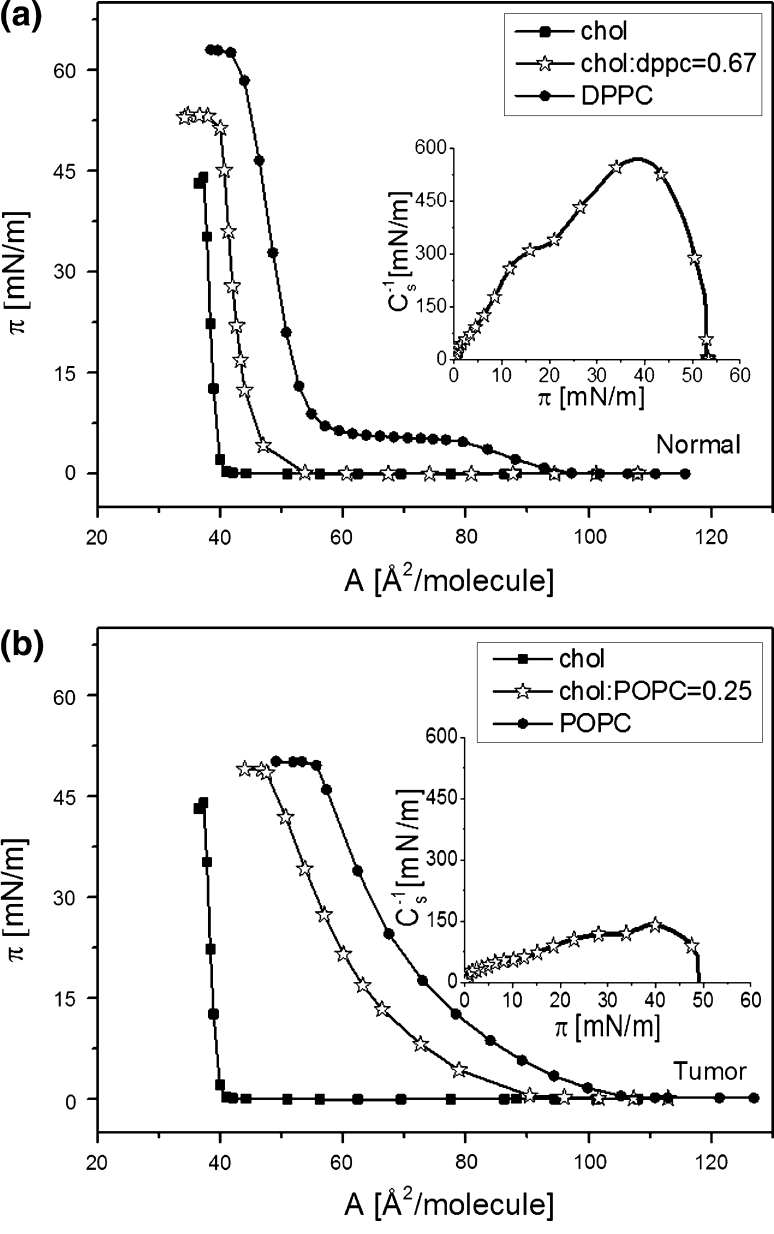
Surface pressure (*π*)–area (*A*) isotherms for binary cholesterol/phosphatidylcholine model membranes. **a** normal, **b** tumor. Insets compression modulus (*C*_s_^−1^)–surface pressure (*π*) dependencies for model membranes

The surface pressure (*π*)–area (*A*) isotherms recorded for binary cholesterol/phosphatidylcholine model membranes are shown in Fig. 9 together with the isotherms for pure components, which are included for the purpose of comparison.

The curves for the respective model systems differ significantly in their shape and position; i.e., the isotherm for normal cellular membrane is steeper and is located at smaller areas compared to that representing a tumor system, which has the character of a liquid-type monolayer.

**Fig. 10:**
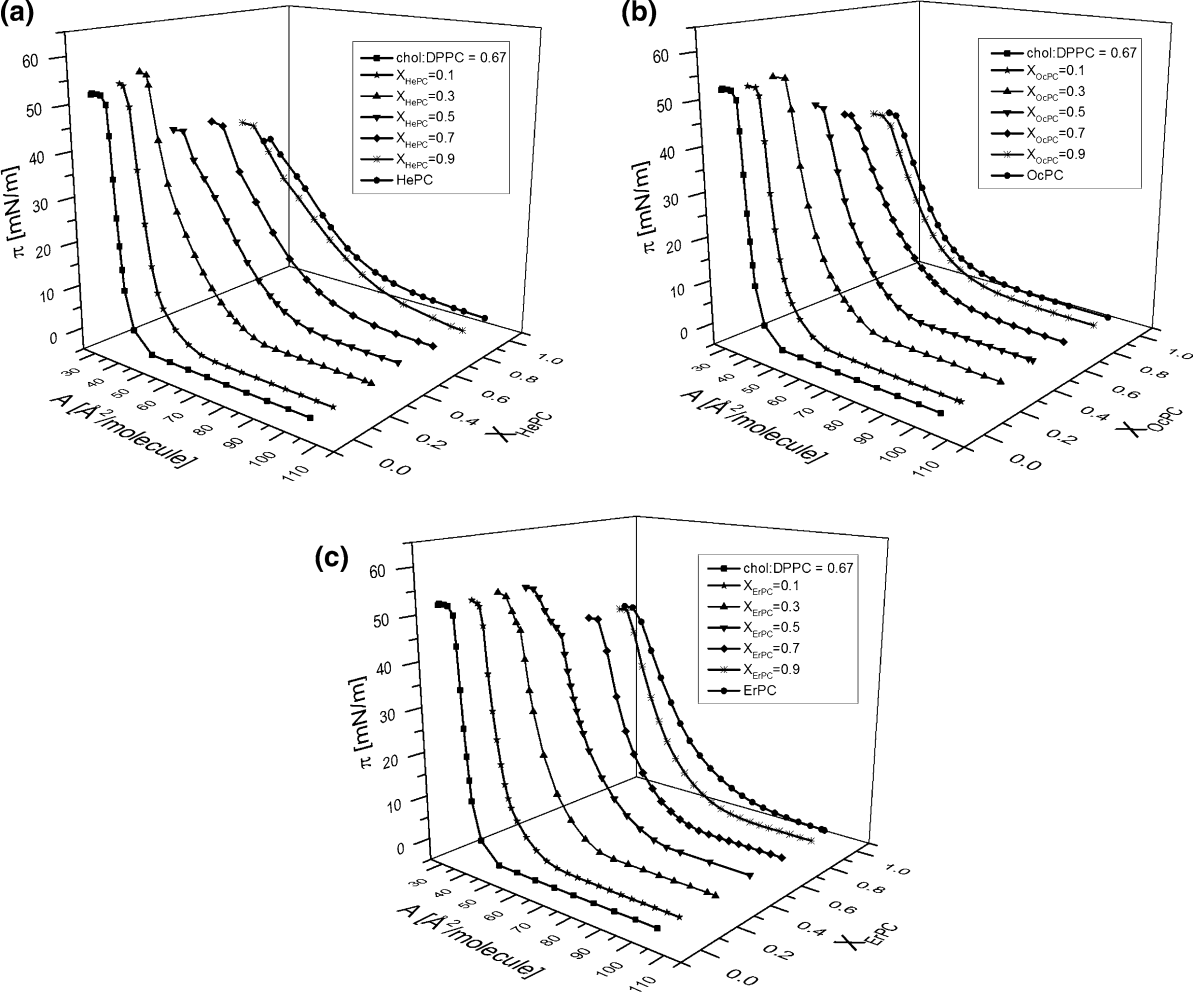
Surface pressure (*π*)–area (A) isotherms for chol/DPPC/APC system

**Fig. 11:**
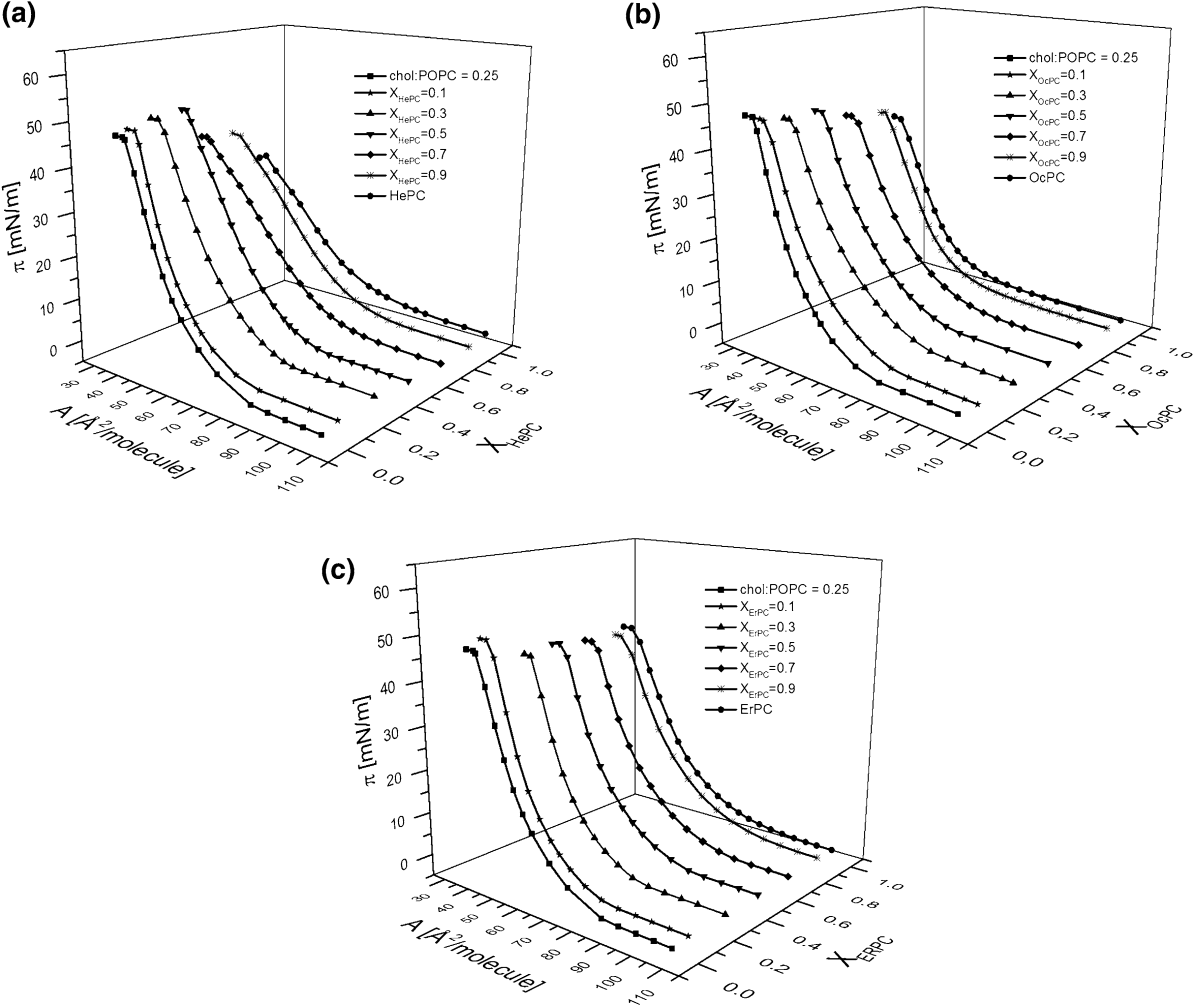
Surface pressure (*π*)–area (A) isotherms for chol/POPC/APC system

The isotherms obtained for ternary cholesterol/phosphatidylcholine/APC monolayers of various drug concentration are presented in Figs. 10 and 11.

**Fig. 12:**
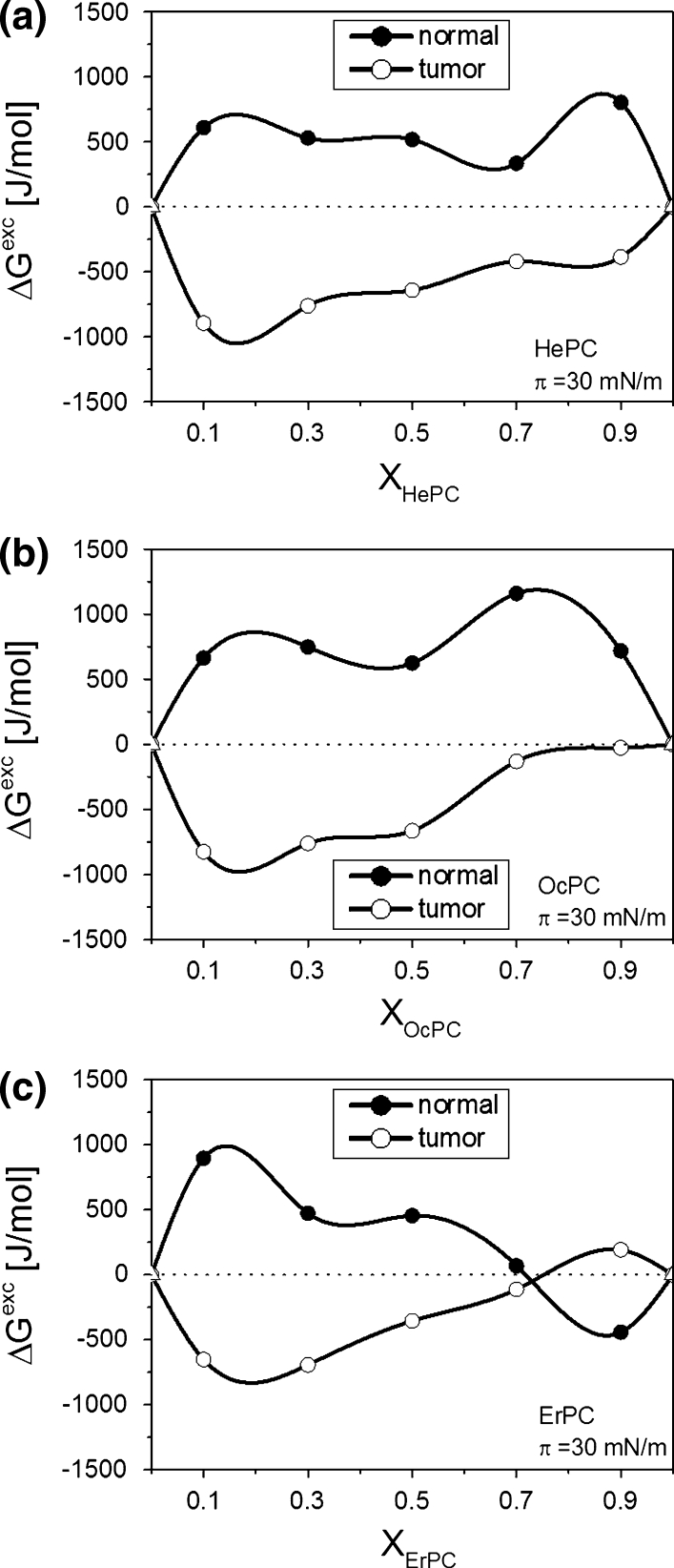
Excess free enthalpy of mixing (Δ*G* ^exc^) versus mixed film composition (X_APCs_) plots for ternary cholesterol/phosphatidylcholine/APC systems at 30 mN/m

The isotherms alone are not very illustrative regarding the influence of APCs on model membranes in contrast to the parameters that can be determined from the experimental isotherm data points, such as the excess free enthalpy of mixing (Δ*G* ^exc^) values. Since the correlation between the properties of lipid monolayers and bilayers has been found in the surface pressure region of 30–35 mN/m (Marsh 1996), the results have been analyzed at *π* = 30 mN/m (Fig. 12).

From Fig. 12 it is evident that APCs affect both membranes, though in opposite ways. Negative values of Δ*G* ^exc^ demonstrate a favorable effect between APCs and model tumor membrane, evidenced by the stronger interactions between the components of ternary cholesterol/POPC/APC monolayers compared to cholesterol/POPC film. In contrast, positive values of Δ*G* ^exc^ for cholesterol/DPPC/APC films show that the interactions are weaker (less attractive or more repulsive) and the film is thermodynamically less stable versus the cholesterol/DPPC system, modeling the normal membrane. Therefore, it may be concluded that the incorporation of APCs to normal cell membranes is thermodynamically unfavorable.

## Discussion

Three synthetic APCs with antineoplastic activities—HePC, OcPC and ErPC—were investigated in Langmuir monolayers formed by membrane lipids, serving as a model of biological membranes. In the first step of our investigations, the above-mentioned APCs were studied in mixtures with single membrane lipids, such as cholesterol, DPPC and POPC. Although it is well known that a biological membrane is a multicomponent system, in order to analyze the results obtained for more complicated mixtures (cholesterol/DPPC and cholesterol/POPC, mimicking membranes of normal and tumor cells), such experiments were necessary.

Studying mixtures of APCs and cholesterol, strong attractive interactions were found between both components in monolayers. The influence of the drugs on cholesterol films was analyzed in a wide range of monolayer compositions and at various surface pressures. It was found that the presence of cholesterol caused the contraction of monolayers from APCs and the mixed film became more condensed upon increasing the proportion of cholesterol. Thermodynamic analysis of APC/cholesterol mixtures proved that the strongest interactions (Δ*G* ^exc^ ~2500 J/mol at 30 mN/m, 20 °C) between the two components occurred when either HePC or OcPC was mixed with cholesterol in a 1:1 proportion. In the case of ErPC, strong interactions with cholesterol were observed within a wide range of mole fractions, without any particular composition showing significantly stronger interactions. Such a strong affinity between components suggests that cholesterol can be of importance in APC incorporation into cell membranes. Namely, molecules of APCs can get “immobilized” with cholesterol, and as a result, only a small amount of “free” drugs can permeate through the biomembrane, exerting its biological activity. Such a lower uptake of antitumor drugs into cellular membranes rich in cholesterol was observed for a synthetic alkyllysophospholipid analogue, edelfosine (Diomede et al. 1990). Although no direct correlation between the cellular level of cholesterol and APC (i.e., HePC) sensitivity was found (Fleer et al. 1993), among various cell lines those possessing the highest phospholipid-to-cholesterol ratio (the epidermoid cancer cell line KB) had the highest capacity for drug uptake, which can be understood based on the results reported here.

Considering phosphatidylcholines, our experiments were concentrated on examining the effect of APCs, being the major lipids of the cellular membrane. Among phosphatidylcholines, two compounds differing in the degree of saturation of the chains were studied, DPPC and POPC. The results for APC/DPPC and APC/POPC mixtures prove that, in general, their interactions are much weaker versus those with cholesterol. Such parameters of interaction, like *A*_12_ or Δ*G* ^exc^ show either positive deviations from ideality (for HePC and OcPC mixed with either with DPPC or POPC) or, in the case of ErPC, small negative deviations at room temperature. From the calculated Δ*G* ^exc^ values it seems that the extent of interactions between APCs with the studied phosphatidylcholines is very similar, independently of the different degree of saturation of the two phospholipids.

To summarize, the above results allow us to conclude that cholesterol can play a more important role in the transport of APCs throughout the biological membrane compared to both investigated phospholipids, which seem to be less important in this aspect.

The affinity of APCs to the studied membrane lipids can also be explained based on the molecular geometry of interacting molecules (Israelachvili 2011). Geometric packing of molecules can be expressed in terms of a dimensionless critical packing parameter, *s*, which depends on the headgroup area (*a*), volume (*V*) and critical length (*l*_c_) of the hydrocarbon chain:

2 $$ s = \frac{V}{{a \cdot l_{\text{c}} }} $$

Values of *a*, *V* and *l*_c_ are characteristic for a given molecule and can be calculated using the following equations:

3 $$ V = (27.4 + 26.9n_{\text{c}} )\, \left[ {{\text{\AA}}^{ 3} } \right] $$

4 $$ l_{\text{c}} = (1.5 + 1.265n_{\text{c}} )\, \left[ {{\text{\AA}}^{ 3} } \right] $$

where *n*_c_ is the number of carbon atoms in the chain; 27.4 and 26.9 Å^3^ are the volumes of the CH_3_ and CH_2_ groups, respectively; 1.265 is the length of the C–C bond; and 1.5 is the radius of the CH_3_ group. The value of *a* is affected by such parameters as the volume of the headgroup, its charge as well as the possibility of hydrogen bond formation and strength; therefore, its value is difficult to estimate. For phosphatidylcholine and cholesterol these values have been calculated to be 71.7 and 19 Å^2^, respectively (Kumar 1991). The value of *s* determines the type of lipid aggregation (Israelachvili 2011).

Our calculations revealed that APCs possess a conical shape—HePC and OcPC, cone; ErPC, truncated cone (see Table 2). In combination with cholesterol of an inverted truncated cone, such an arrangement of opposite molecular geometries ensures favorable packing of APCs and cholesterol, resulting in strong attractive intermolecular interactions, observed in Fig. 5a. These results can also explain the lack of hemolytic activity of ErPC in comparison to other APCs (Jendrossek et al. 2003). Namely, a clear minimum at a 1:1 molar fraction in Δ*G* ^exc^ = *f*(*X*_HePC/OcPC_) graphs implies that up to this proportion HePC and OcPC are bound with cholesterol in the form of complexes and no excess of molecules of these alkylphoshocholines remains at the surface. However, upon exceeding the amount of these drugs above the 1:1 proportion, “free” (unbound with cholesterol) molecules of HePC and OcPC are present, which are responsible for the hemolytic activity. The system ErPC/cholesterol behaves differently; i.e., practically within the whole mole fraction (except for the region of very low ErPC content) drug molecules strongly interact with cholesterol. This allows us to understand the difference between ErPC and other investigated APCs in terms of hemolysis.

**Table 2:** Molecular packing parameters of membrane lipids and APCs

Component	Parameters				
	*a* (Å^2^)	*l*_*c*_ (Å)	*V* (Å^3^)	*s*	Shape
Lipids					
Cholesterol	19	17.250	400	1.22	
DPPC	71.7	20.475	847	0.57	
POPC	71.7	20.475	910	0.619	
APCs					
HePC	71.7	20.475	430.9	0.29	
OcPC	71.7	23.005	484.7	0.29	
ErPC	71.7	24.400	582.9	0.33	

*s* Critical packing parameter; *a* headgroup area, the volume; *V* volume of hydrocarbon chain; *l*_*c*_ critical length of hydrocarbon chain

On the other hand, phosphatidylcholine molecules are of a truncated cone shape. Therefore, a combination with conically shaped APCs does not meet the condition of geometrical complementarity, which explains weak APC/DPPC(POPC) interactions with a tendency to phase separation.

The results obtained for systems imitating natural membranes prove that the insertion of APCs modifies the properties of both model systems, though in a completely different way. The monolayers mimicking tumor and normal cell membranes are of different molecular organization due to different compositions of their component lipids. Cholesterol/DPPC film is more ordered and condensed than the cholesterol/POPC system as shown by higher compression modulus values found for normal cell model membrane compared to the tumor model system (Fig. 9, inset). Studies of mixed monolayers of cholesterol/DPPC (Dynarowicz-Latka et al. 2002) and cholesterol/POPC (Jurak 2013) prove that the affinity of cholesterol to DPPC is stronger than that to POPC. Therefore, the insertion of APCs molecules into a more liquid and less ordered cholesterol/POPC monolayer is much easier than the incorporation of drug molecules into cholesterol/DPPC film. This enables us to conclude that the membrane of normal cells is a natural barrier, preventing drug molecules from penetrating into healthy cells and explains the high selectivity of APCs.
